# Ozone in Spain's National Parks and Protected Forests

**DOI:** 10.1100/tsw.2007.8

**Published:** 2007-03-21

**Authors:** María J. Sanz, Francisco Sanz, Vicent Calatayud, Gerardo Sanchez-Peña

**Affiliations:** ^1^ Fundación C.E.A.M., Charles R. Darwin 14, Parc Tecnològic, 46980 Paterna, Valencia, Spain; ^2^ Servicio de Protección contra los Agentes Nocivos, DGB, MIMAM, Gran Vía San Francisco 4, Madrid, Spain

**Keywords:** air pollution, ozone, passive samplers, Mediterranean, national parks, Spain

## Abstract

In general, it is difficult to measure air pollutant concentrations in remote areas, as they are mostly national parks and protected areas. Passive samplers provide an accurate and inexpensive method for measuring cumulative exposures of different air pollutants. They have been used to collect ozone data in both laboratory and field at different geographical scales. The objective of the present study is to fill the knowledge gap regarding air quality in remote areas of Spain, such as national parks and protected areas. Because there were no systematic data sets on the main air pollutants that could affect these areas, an air quality measurement network was established between 2001 and 2004 on 19 locations inside Spanish national parks and protected areas. The data collected suggest that ozone levels in mountainous areas are high enough to affect sensitive vegetation. Most of the locations registered moderate-to-high ozone levels, with important interannual variability. Altitudinal ozone gradients were observed in most of the parks with complex topography due to the establishment of local circulations that incorporate polluted air masses from polluted airsheds or even long-range transport (i.e., Canary Islands). Different latitude-dependent, yearly cycles were also observed, showing two, one, or no clear peaks depending on the region. These findings extend to the most southerly locations, except in the Canary Islands, where pollution transported from other regions in the upper transport layers probably led to the high concentrations observed.

## INTRODUCTION

At present, tropospheric ozone is considered by far the most important toxic air pollutant for plants worldwide[1,2]. In certain areas, tropospheric ozone, acting through chronically enhanced regimes[3], is regarded as the air pollutant with the most injurious potential for vascular plants. This is particularly relevant in view of the environmental scenarios foreseen for the remainder of this century[4,5]. In fact, tropospheric ozone has been recognized as a potentially important factor within "global change"[6,7], perhaps even able to curtail the carbon sink strength of plants and, therefore, of ecosystems to an extent similar to stimulation by elevated $CO_2$ at the global scale[8,9]. In addition, tropospheric ozone pollution has shifted from a regional to a global issue following the confirmation of intercontinental $O_3$ transport[10,11,12]. Ozone regimes are predicted to remain high or even increase (in terms of background $O_3$ levels), contributing to global warming during the course of the 21$^{st}$ century[13,14]. Depending on the region[15], such trends will be driven by the continued anthropogenic release of accompanying pollutants (e.g., $CH_4$, CO[16]) and precursors (e.g., NOx). Spatial and temporal $O_3$ patterns at regional level may be complex and depend not only on precursor emissions, but also on mesoscale topography and three-dimensional atmospheric dynamics[17,18,19].

Reports by the European Environmental Agency[20,21] suggest that the Mediterranean region is the highest ozone risk area in Europe; it registered the highest number of exceedances of ozone warning/alert thresholds between 1995 and 2004[21]. In the last decades, the Mediterranean region has experienced an important increase in population, industry, and traffic, especially in coastal areas. Nevertheless, despite some point sources of primary pollutants, $O_3$ is the main pollutant in the area since under Mediterranean climatic conditions, strong photochemical activity is experienced[22]. Moreover, the meteorology in the Mediterranean basin is driven by marked mesoscale circulations that are strongly linked to the complex topography[16,18,19,23,24]. It is now known that these mesoscale circulations are consistent features of the Mediterranean climatic system, and that the ozone cycles observed in the Western Mediterranean basin depend on the topographic location and can show large differences within radii of tens of kilmeters[19,25]. In coastal valleys, ozone concentrations and injuries followed similar complex spatial patterns[23,26,27] on all 83 southwestern remote sites (France, Italy, Switzerland, and Spain) belonging to the ICP-Forest Intensive monitoring Plots (IMPs): the April-September average daily ozone concentrations (2000–2001) decreased with ascending latitude and increased with altitude. Such patterns were confirmed for the years 2002 and 2003, with higher concentrations in 2003, by ICP-Forest across Europe[28,29].

Practically half of all European endemisms are from Spain, and most of them are located in protected areas like national parks or natural protected areas. Photo-oxidants, and especially ozone, could be a potential risk to these plants[24]. Up to now, most studies on ozone effects have been focused on crops and forest species, including surveys in protected areas (e.g.[30]), but little work has been done on endemic or rare plant species because their habitats are mostly in rural or remote areas where ozone information is scarce.

In general, it is difficult to measure air pollutant concentrations in remote areas, as they are mostly national parks and/or protected areas. Passive samplers provide an accurate and inexpensive method for measuring cumulative exposures of different air pollutants[31]. They have been used to collect ozone data in both laboratory[32,33] and field studies at different scales, from local to continental[26,28,34,35,36,37,38], demonstrating that passive samplers can function with high accuracy and precision under a variety of ambient conditions. However, few of these studies are being carried out in national parks or protected areas[36,39,40], where they may be an extremely interesting tool for managers.

The objective of the present study is to fill the gap in our knowledge about air quality in remote areas of Spain, such as national parks and protected areas, because of the lack of systematic data sets on the main air pollutants that may affect these areas.

## MATERIAL AND METHODS

### Study Area

A rural air quality measurement network was established between the spring of 2001 and the spring of 2004 at 19 locations within the boundaries of most of the Spanish national parks and protected areas under the supervision of the National Parks Department (Fig. 1, Table 1).

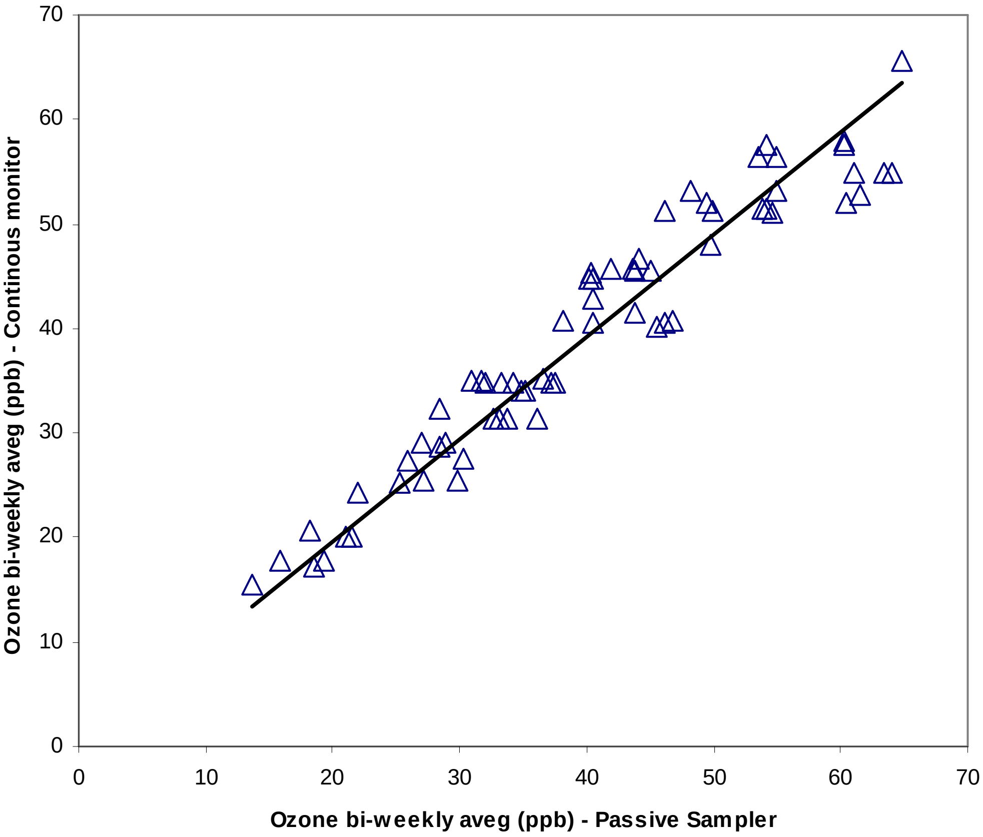

**FIGURE 1**. Ozone measured by passive samplers vs. measured with active monitors (Dasibi Model 1008 RS) throughout the study for all sampling periods. The regression equation: $y = 0.901x + 3.51$ $r^2 = 0.911$ (being the individual stations: Gandia, $y = 0.879x + 4.19$ $r^2 = 0.914$; Benifaió, $y = 0.736x + 5.29$ $r^2 = 0.912$; Morella, $y = 0.677x + 15.52$ $r^2 = 0.634$).

## Ozone Monitoring

Passive samplers from Ogawa & Co. (Pompano Beach, FL) were selected for the study. In the presence of ozone, the nitrite is oxidized into nitrate, and the filters are analyzed for total nitrate concentration following the exposure period. From the total nitrate concentration, a biweekly average ozone concentration (ppbv) is calculated as follows[41]:

$$E_{sample} = [(NO_3^-)* \text{Vol} * 6.572 \times 10^3] * R^{-1} \quad (1)$$

$$E_{\text{blank}} = [(NO_3^-)* \text{Vol} * 6.572 \times 10^3] * R^{-1} \quad (2)$$

$$E_{net} = [E_{sample} - E_{\text{blank}}] \quad (3)$$

$$\{O_3\} = [E_{net} * t^{-1}] \quad (4)$$

**TABLE 1**
**Coordinates and Altitude of the Different Sampling Locations**

| Park Name | Location Number | Site Name | Longitude | Latitude | Altitude (msm) |
|---|---|---|---|---|---|
| Ordesa N.P. | 1b | OR-PARADOR TORLA | 00º 05'45.1"W | 42º 39'19.3"N | 1,204 |
| | 1a | OR-CAÑON AÑISCLO | 00º03'25.3"E | 42º33'41.5"N | 1,105 |
| Aigüestortes N.P. | 2 | AIGÜESTORTES | 00º54'04.6"E | 42º33'32.4"N | 2,320 |
| Picos de Europe N.P. | 3b | P.E.-BUFERRERA | 04º 58' 54.6"W | 43º 16' 23.4"N | 1,086 |
| | 3a | P.E.-DEP. COVADONGA | 05º 03' 29.4"W | 43º 18' 37.4"N | 300 |
| Cabañeros N.P. | 4 | CABAÑEROS | 04º18'45.0"W | 39º18'15"N | 634 |
| Doñana N.P. | 5b | DOÑ-MANECORRO | 06º29'36.7"W | 37º7'21,1"N | 20 |
| | 5a | DOÑ-ACEBUCHE | 06º34'3.3"W | 37º2'44,1"N | 15 |
| Garajonay N.P. | 6 | GARAJONAY-ALT CHERELEPIN | 17º 15' 30.0"W | 28º 06' 50.0"N | 1,300 |
| Caldera de Taburiente N.P. | 7b | LA PALMA-ESPIGON | 17º 53' 01.4"W | 28º 45' 11.1"N | 2,400 |
| | 7a | LA PALMA-LOMO LAS CHOZAS | 17º 51' 43.2"W | 28º 41' 49.8"N | 1,260 |
| Cañadas del Teide N.P. | 8b | TEIDE-MONT RAJADA | 16º35'44.6"W | 28º15'58,1"N | 2,498 |
| | 8a | TEIDE-CAÑADA | 16º33'03.4"W | 28º16'30,1"N | 2,111 |
| Sierra Nevada N.P. | 9c | S.N.-LA RAGUA | 03º 01' 13.9"W | 37º 06' 55.7"N | 2,133 |
| | 9b | S.N.-CORTIJUELA | 03º 28' 07.3"W | 37º5'09.8"N | 1,763 |
| | 9a | S.N.-PUENTE PALOS | 03º 15' 25.6"W | 37º 06' 35.2"N | 1,713 |
| Quintos de Mora S.P.L. | 10 | QUINTOS DE MORA | 04º04'58.9"W | 39º26'14.6"N | 1,114 |
| Lugar Nuevo S.P.L. | 11 | LUGAR NUEVO | 04º04 50.9"W | 38 10 49.8"N | 640 |
| La Almoraima S.P.L. | 12 | LOS ALCORNOCALES | 05º26'02.2"W | 36º17'10.8"N | 87 |

N.P. = National Park, S.P.L. = State Protected Land ("Finca del Estado").

where $E$ is ppbv, $\{O_3\}$ is the ozone mixing ratio ($ppbv^{-1}$), $t$ is the exposure time (h), $R$ is the collection factor (21.6 cc/min), Vol is the extraction volume (ml), and ($NO_3^-$) is the nitrate determined by analysis (μg $ml^{-1}$). Previous studies with these samplers have shown that differences in barometric pressure and temperature do not have a significant effect on diffusivity (i.e., deposition rates) and conversions of nitrite to nitrate[33,41].

Two passive samplers were housed in a PVC pipe rainshield and hung from a 2.5-m metal stake in open areas. Two blanks were shipped with the samplers and were kept in the office in each park during the sampling period. The ozone passive samplers prepared for each sampling period were calibrated against Dasibi 1008RS ozone monitors at two locations in the Valencia Community Air Quality Network (Morella, Gandía) and at the CEAM OTC experimental field (Benifaió). The results of these calibrations were used for correcting ozone concentrations for the remaining passive samplers in the sampling network.

When the sites were established, a short training course and an operation manual were given to all the park agents involved in the study and field sheets were provided on each shipment. The agents placed and collected the samplers about every 15 days, and they sent them to and from the laboratory by express mail within 48 h for their analysis. Once in the laboratory, the bags were opened and the filters removed from the sampler in an ozone-free chamber. Analysis was conducted by the analytical chemistry laboratory in Foundation CEAM. Standardization of the field equipment and all the procedures was a decisive factor in achieving consistent results.

### Data Analysis

The 2-week average ozone concentrations were calculated for each passive sampler location (ppbv). Sampler precision corresponded to the percent difference between replicate samplers at each site. Sampler accuracy was determined by comparing passive sampler values with the continuous analyzer hourly averages for identical sampling periods. By plotting both 2-week averages (from the monitors and the passive samplers), a linear regression equation was obtained (SPSS+), which was later used for corrections. Ozone passive sampler values were plotted against elevation to detect whether there was any relationship for the 3-year sampling periods (SPPS+).

## RESULTS AND DISCUSSION

### Validation of Ozone Passive Samplers

The mean ozone concentration determined from the blanks during the sampling period was 3–4 ppbv. The precision of the pairs of ozone samplers was estimated as 4 ppbv (maximum 7 and minimum 3), with an average variation coefficient of 12% during the study period (maximum 20% and minimum 5%). Linear regression analysis was used to compare ozone measurements by passive sampler and by continuous monitoring (Dasibi 1008RS). The regression equation gave a slope close to 1 (0.901, $r^2 = 0.911$) with a y-intercept of 3.5 (Fig. 2) for all stations pooled and was evenly distributed over the study period, which can be considered insignificant given the precision of the samplers in most of the cases. When the concentrations of the three locations are plotted separately, the $r^2$ is lower on locations where strong winds are frequent. Data were corrected using the equation (Fig. 2) to produce the final results.

### Temporal Evolution of Ozone Concentrations

During the study period considered, two main ozone peaks were observed at almost all locations for at least 1 year: one in late spring and one in midsummer with a temporal decay in July (Fig. 3). The same type of behavior has been observed in other monitoring networks on the east coast of Spain where only electronic monitors[19] or passive samplers[35] are used. Thus, passive ozone sampling appears to be a useful tool for studying the annual temporal variation in ozone concentrations over the year when 2-week periods are considered. For all the passive sampler locations, minimum ozone values are found during fall and winter, as expected. Maximum values appeared in spring and summer. During the study period, values ranged from 7 ppbv in Picos de Europa in winter to 102 ppbv in Quintos de Mora in late summer.

Ozone variability among years is also observed (Fig. 3). Among the years included in this study, the second half of both 2001 and 2002 showed by far the highest ozone concentrations for all locations in the central and southern part of the Iberian Peninsula (Quintos de Mora had a maximum 102 ppbv in 2002), and to a lesser extent in the northwest.

In the Canary Islands parks, except for the highest elevation location in Las Cañadas del Teide N.P., there are no major differences among years. The largest minimum values are found in La Caldera de Taburiente (La Palma) with 35 ppbv and Las Cañadas del Teide (Tenerife) with up to 39 ppbv, both for the second half of 2001. The largest maximum values are found in La Caldera de Taburiente (La Palma), with 76 ppbv, and in Las Cañadas del Teide (Tenerife), with up to 80 ppbv both for the first half of 2002.

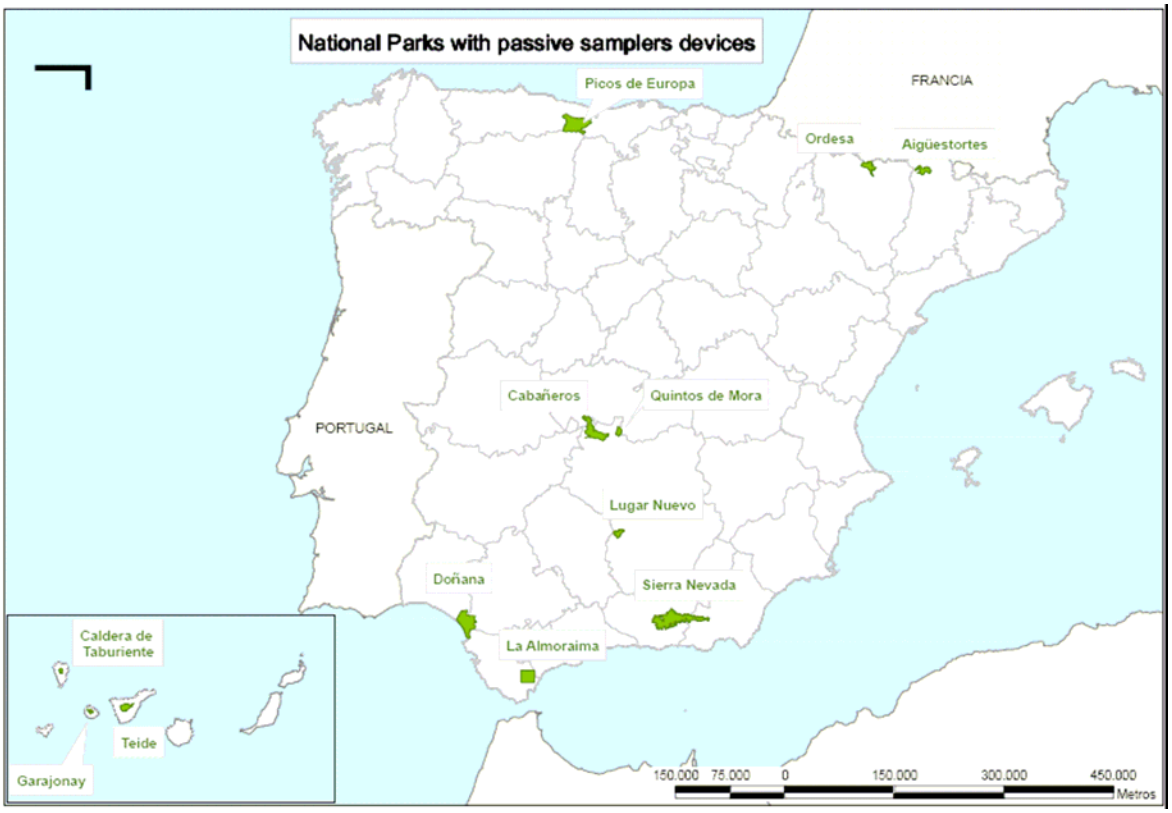

**FIGURE 2.** National parks and State protected lands within the network.

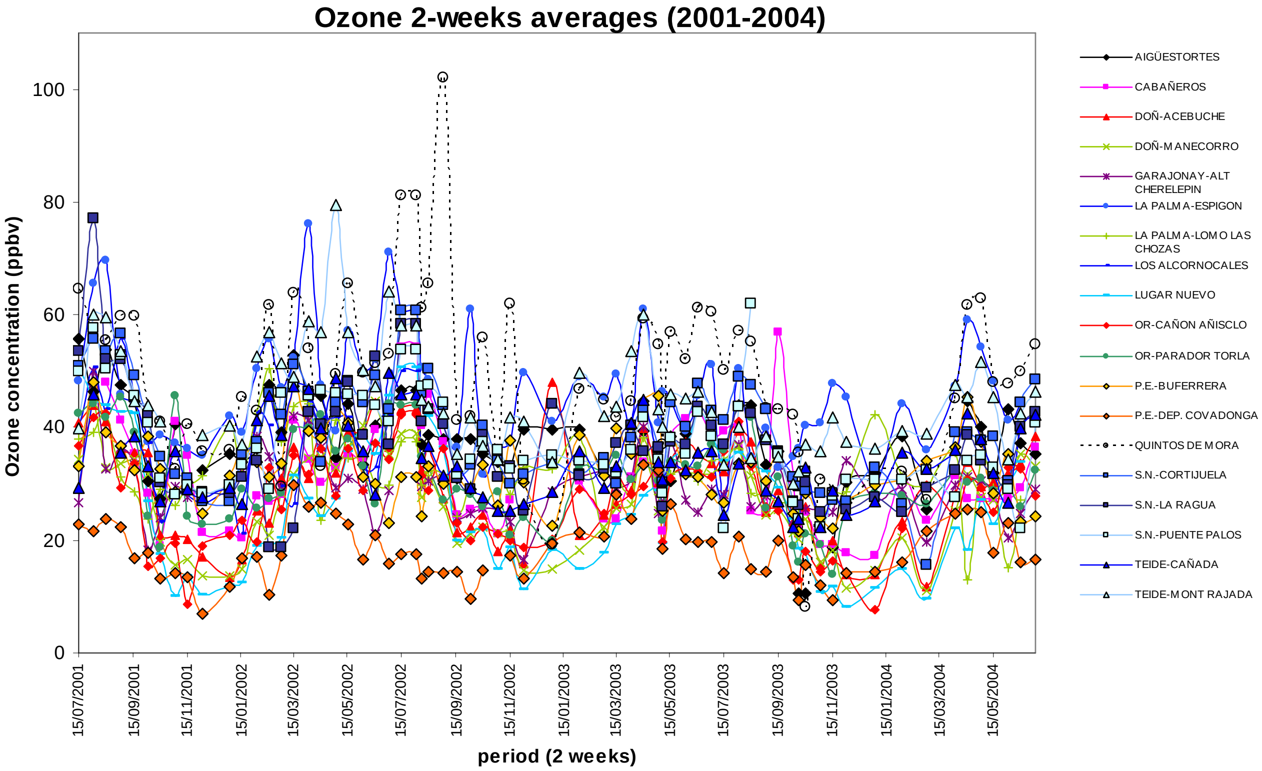

**FIGURE 3**. Biweekly averages for the 3 years (July 2001–July 2004) for all sites.

## Geographical Patterns and Altitudinal Gradients

Data from sampling periods between 1 April and 30 September were used to compute the seasonal average; this is considered the growing season for trees when calculating ozone critical loads in forests[4]. If we select the year 2002, a high ozone year, the observed spatial geographical pattern at Peninsular scale indicates that, when the average of April–September ozone concentrations is considered, the Eastern Iberian Peninsula has higher ozone values than the western part (Fig. 4). The Canary Islands have to be considered separately; most of the parks showed elevated ozone seasonal averages.

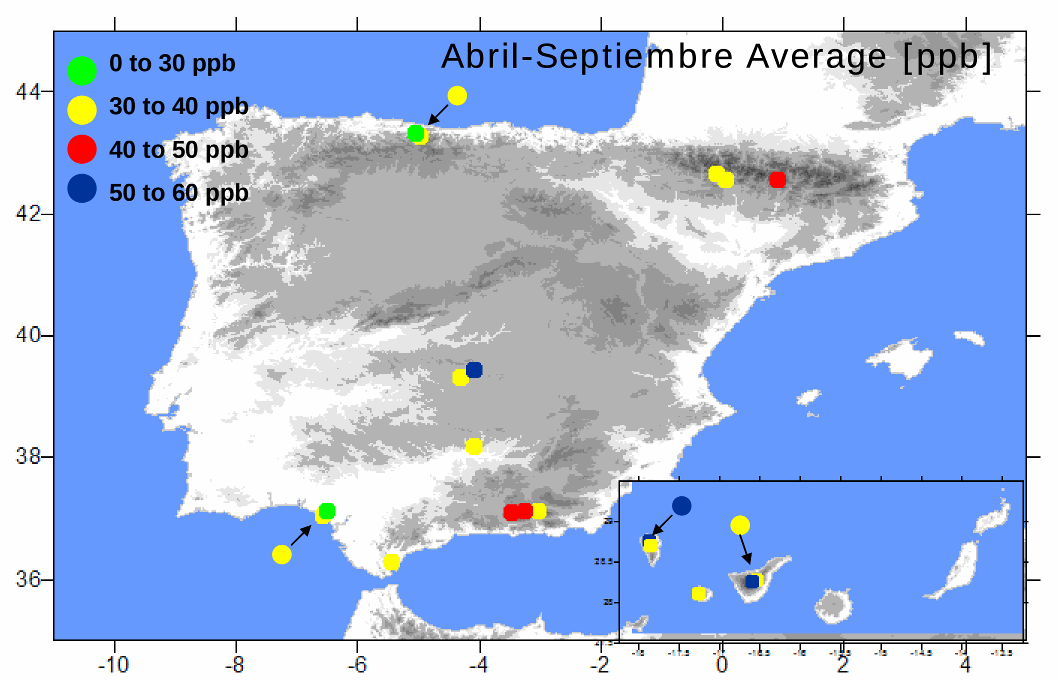

**FIGURE 4**. Maps with ozone seasonal (April–September) averages for 2002 (ppbv).

In Fig. 5, the ozone maximum and minimum and the yearly biweekly averages were plotted for each park for 2002. In cases where ozone was measured on an elevational gradient within a park, the arrows indicate a tendency for ozone concentration change as elevation increases (see Fig. 5, for 2002). Exceptions were the Sierra Nevada N.P. where the mid-altitude locations had the highest ozone concentrations probably due to a different exposure (N exposure, more influenced by the plume of Granada), and Doñana N.P. where the locations have a minimum difference in altitude due to the lack of topography of the Park.

When the ozone biweekly averages for the three yearly periods, starting in mid 2001, are plotted against altitude, excluding Quintos de Mora where the most extreme ozone levels were observed, the correlation coefficients observed were always greater than $r^2 = 0.6$, with very similar slopes and intercepts (Fig. 6). In the same line, altitudinal dependency is being widely found on European forest sites[27].

The high ozone values observed at high elevations probably result from the fact that such locations are influenced most of the time by the reservoir layers generated by valley recirculation[16,18] or long-range transport[12]. Thus, the decoupling between upper and lower stations may occur more often in winter when the altitude of the inversion layer is lower, whereas during the warmer seasons, a combination of two types of processes may be responsible for the gradients observed: (1) photochemical production with increasing distance from the source of precursors combined with local transport of the air

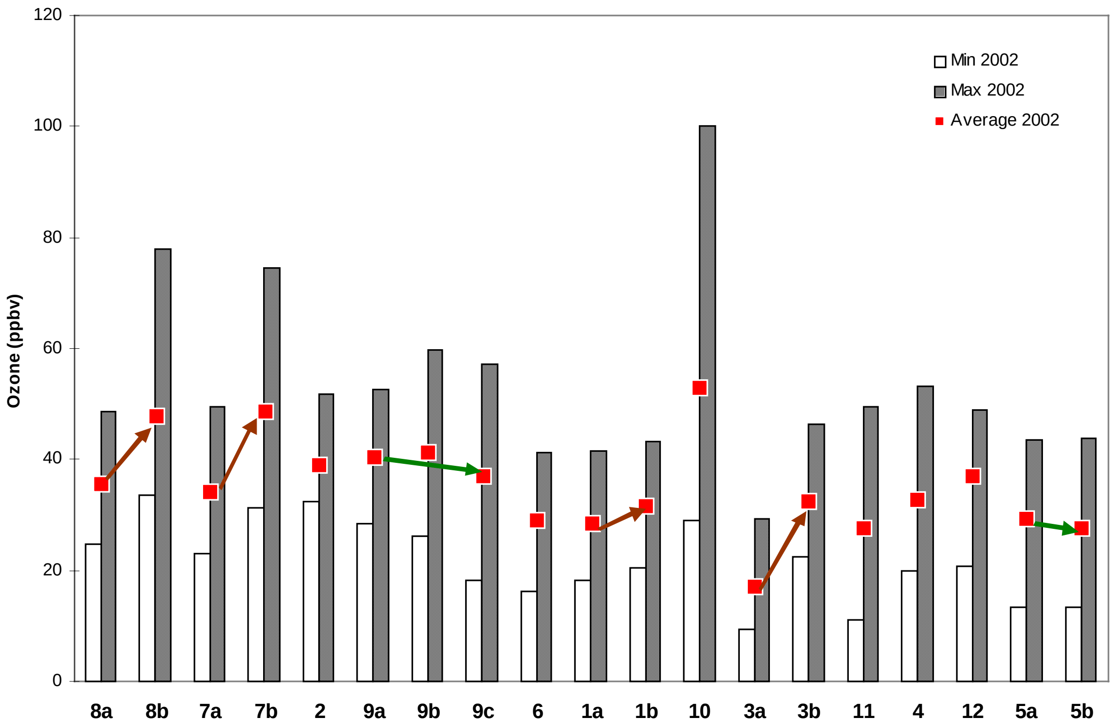

**FIGURE 5**. Average, maximum and minimum ozone concentration (biweekly periods) for 2002, the year with highest ozone among the ones included in the paper. Parks ordered by maximum altitude and sampling locations ordered by altitude (m) with in the park.

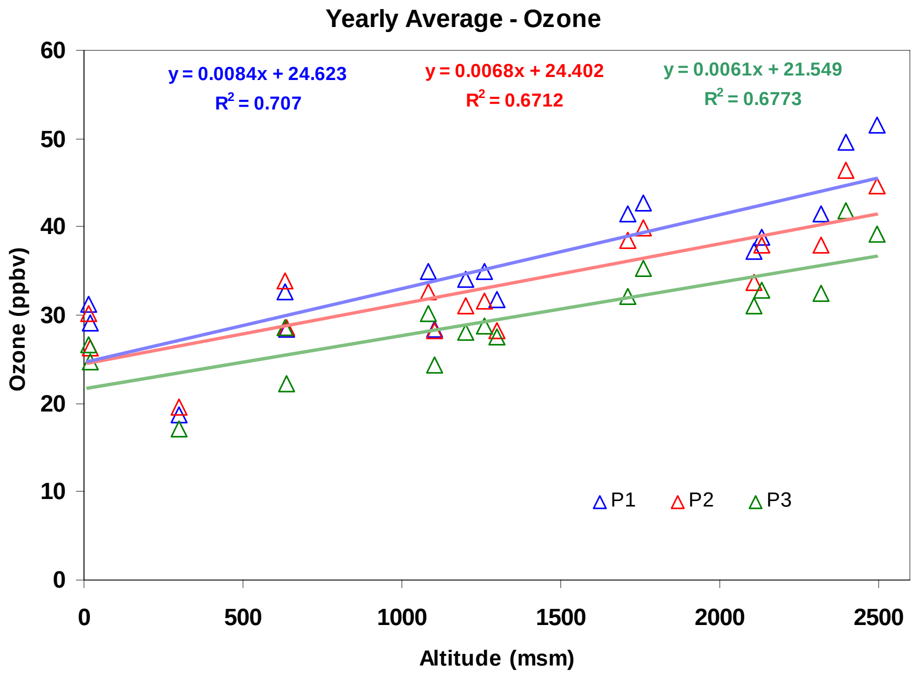

**FIGURE 6**. Relationship between the ozone yearly average (ppbv) and the altitude (msm) of the sampling location for the three sampling periods (P1 = July 2001–June 2002; P2 = July 2002–June 2003; P3 = July 2003–June 2004). Note: Quintos de Mora and Los Alcornocales were not considered due to the extremely high concentrations in the first case for few periods in summer and the few data available in the later case.

mass within the basin as a result of the recirculation, and (2) the existence of reservoir layers aloft generated during previous days or long-range transport. Topography drives the circulation patterns at the local level, with the strength of the up-slope winds dependent on solar radiation heating of the slopes and the properties of the surface during the day, which could explain the altitudinal gradients in the different parks (Fig. 5). At night, down-slope drainage flows are established[19].

## Averaged Yearly Cycles

When averaging the biweekly values per month for the whole study period (July 2001–July 2004), different seasonal cycles can be observed (Fig. 7): (a) yearly cycles with two clear peaks, one in spring and one in summer, are seen in the north where it seems the summer peak is higher on high locations; (b) yearly cycles with a clear peak in summer; and (c) cycles with no clear peaks or more than two peaks in the Canary Islands. Cases (a) and (b) are probably generated from local to regional processes, whereas case (c) can be attributed more to transported layers.

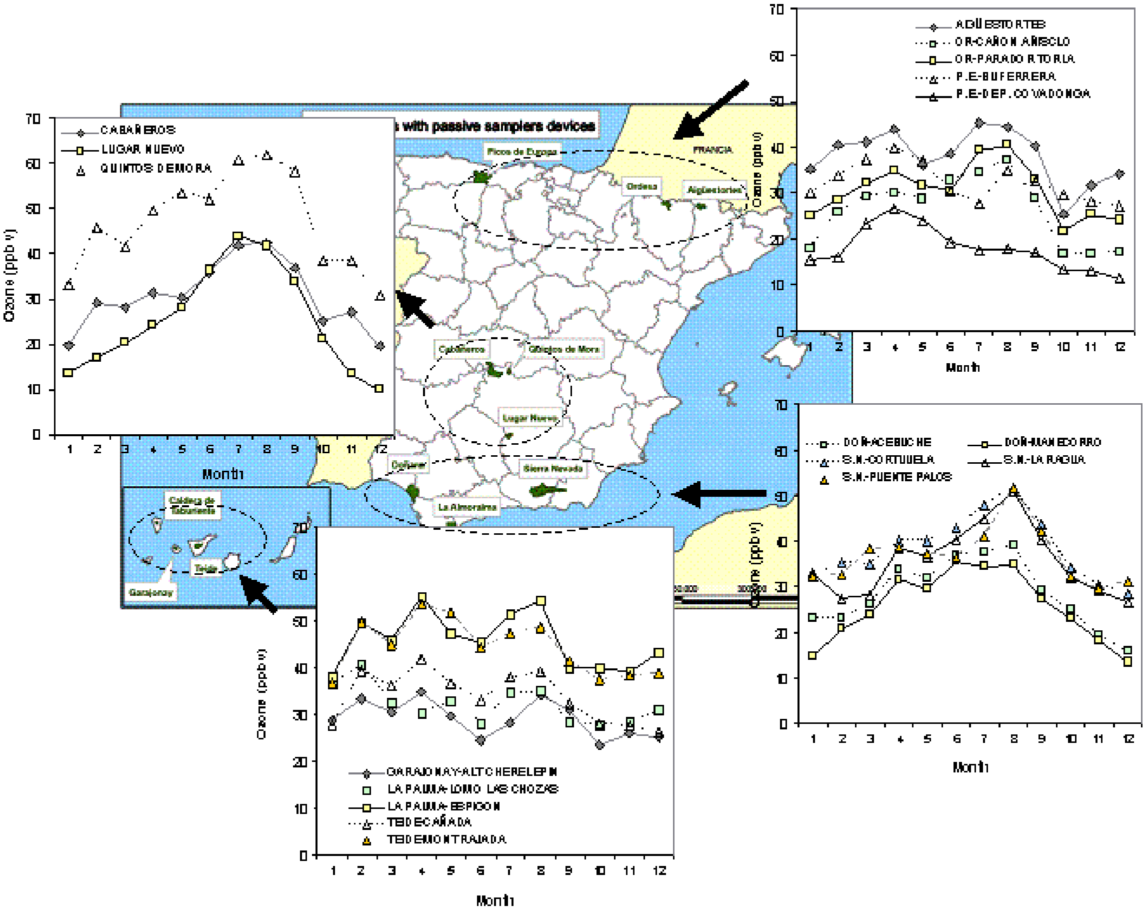

**FIGURE 7**. Yearly averaged cycles in different regions for the study period (July 2001–July 2004).

## CONCLUSIONS

Most of the national parks and state forests in Spain registered moderate-to-high ozone levels, with important interannual variability. Maximum values were registered in spring and summer, whereas minimum values appeared in winter.

As in other studies, altitudinal ozone gradients were observed in most of the parks with complex topography, due to the existence of local-to-regional mesoscale circulations that transport polluted air masses from polluted airsheds or, in some cases, long range transport (i.e., Canary Islands). Exception to the rule was the Sierra Nevada Park, were the mid-elevation site presented the highest values due to different exposure (N) more under the influence of precursors from the city of Granada.

Different latitude-dependent yearly cycles were observed, with two, one, or no clear peaks from north to south. This includes the most southerly locations in the Canary Islands, where pollution transported from other regions in the upper layers probably dominates.

## ACKNOWLEDGMENTS

The authors thank the Generalitat Valenciana and Bancaja for their continuous support to Fundación CEAM. We are indebted to all the national park agents that helped us to select the sampling sites and participated in the field work by collecting the samplers and sending them to our lab during the survey. Passive sampler calibrations and development were done under ODOPAS project (REN2002-04337-CO2-O2). The surveys were made possible due to the support of the DGB of the Ministry of Environment and the National Parks Authority.
